# Successful implementation of isoniazid preventive therapy at a pediatric HIV clinic in Tanzania

**DOI:** 10.1186/s12879-020-05471-z

**Published:** 2020-10-07

**Authors:** Olivia F. Hunter, Furaha Kyesi, Amrit Kaur Ahluwalia, Zeinabou Niamé Daffé, Patricia Munseri, C. F. von Reyn, Lisa V. Adams

**Affiliations:** 1 Dartmouth College, Hanover, NH 03755 USA; 2 New York, USA; 3 Ministry of Health, Community Development, Gender, Elderly, and Children, Dodoma, Tanzania; 4 Muhimbili University of Health and Allied Sciences, Dar es Salaam, Tanzania; 5 Dartmouth-Hitchcock Medical Center, Lebanon, NH 03756 USA; 6 Geisel School of Medicine at Dartmouth, Hanover, NH 03755 USA

**Keywords:** HIV, Pediatric, Tuberculosis, TB, Tanzania, Isoniazid, IPT

## Abstract

**Background:**

In accordance with international guidance for tuberculosis (TB) prevention, the Tanzanian Ministry of Health recommends isoniazid preventive therapy (IPT) for children aged 12 months and older who are living with HIV. Concerns about tolerability, adherence, and potential mistreatment of undiagnosed TB with monotherapy have limited uptake of IPT globally, especially among children, in whom diagnostic confirmation is challenging. We assessed IPT implementation and adherence at a pediatric HIV clinic in Tanzania.

**Methods:**

In this prospective cohort study, eligible children living with HIV aged 1–15 years receiving care at the DarDar Pediatric Program in Dar es Salaam who screened negative for TB disease were offered a 6-month regimen of daily isoniazid. Patients could choose to receive IPT via facility- or community-based care. Parents/caregivers and children provided informed consent and verbal assent respectively. Isoniazid was dispensed with the child’s antiretroviral therapy every 1–3 months. IPT adherence and treatment completion was determined by pill counts, appointment attendance, and self-report. Patients underwent TB symptom screening at every visit.

**Results:**

We enrolled 66 children between July and December 2017. No patients/caregivers declined IPT. Most participants were female (*n* = 43, 65.1%) and the median age was 11 years (interquartile range [IQR] 8, 13). 63 (95.5%) participants chose the facility-based model; due to the small number of participants who chose the community-based model, valid comparisons between the two groups could not be made. Forty-nine participants (74.2%) completed IPT within 10 months. Among the remaining 17, 11 had IPT discontinued by their provider due to adverse drug reactions, 5 lacked documentation of completion, and 1 had unknown outcomes due to missing paperwork. Of those who completed IPT, the average monthly adherence was 98.0%. None of the participants were diagnosed with TB while taking IPT or during a median of 4 months of follow-up.

**Conclusions:**

High adherence and treatment completion rates can be achieved when IPT is integrated into routine, self-selected facility-based pediatric HIV care. Improved record-keeping may yield even higher completion rates. IPT was well tolerated and no cases of TB were detected. IPT for children living with HIV is feasible and should be implemented throughout Tanzania.

## Background

Tuberculosis (TB) represents an enormous global disease burden, especially for people living with human immunodeficiency virus (HIV) [1, 2]. Rates of TB disease are particularly high in children living with HIV [3, 4]. Deaths from HIV/TB co-disease can be reduced by preventing or treating latent TB infection [5, 6]. Standard HIV antiretroviral therapy (ART) alone is insufficient in preventing pediatric TB in high TB burden countries [7].

Consequently, the World Health Organization (WHO) has recommended expanded delivery of isoniazid preventive therapy (IPT) to reach those at greatest risk for progressing to TB disease [3], including children living with HIV. In this population, IPT has been shown to reduce mortality by 50% and incidence by more than 70% in high TB-burden countries [8]. Due to the low likelihood of drug-drug interactions, evidence of effectiveness, and low cost, the WHO recommends that people living with HIV, including children older than 12 months, receive at least 6 months of IPT as part of comprehensive HIV care [9].

Despite international endorsement [10], IPT enrolment and completion has remained low, especially in low-income countries with a high TB burden [11]. In South Africa and Uganda, for example, IPT uptake was found to be as low as 15% even when available [12]. Barriers to uptake include fear of drug resistance, difficulty of screening for TB due to lack of access to TB diagnostic tests, negative or unfriendly interactions between healthcare workers and parents, stigma, and fears of side effects [13–17]. To support IPT rollout, the WHO has created a simplified screening algorithm based on clinical symptoms that increases cost-effectiveness of TB screening [18, 19]. Furthermore, provision of choice of IPT delivery models, such as community-outreach versus facility-based care, combining IPT and ART refills into single visits, and providing flexible appointment schedules have been associated with rates of completion and adherence of over 80% [20–22].

Tanzania is one of the 30 high burden countries for TB and TB/HIV co-disease; an estimated 40,000 Tanzanians developed HIV-associated TB in 2018 [23]. While the Ministry of Health in Tanzania has officially recommended IPT for children living with HIV since 2009 [24], implementation is still not the standard of care at many healthcare facilities [25]. This study sought to determine whether patient-selected IPT delivery coordinated with ART delivery would yield high adherence and treatment completion in children living with HIV in Dar es Salaam.

## Methods

We conducted a prospective cohort study of patient-selected IPT delivery models at the DarDar Pediatric Program clinic (DPP) in Dar es Salaam. The clinic provides routine outpatient HIV care for children aged 15 and below together with their parents/caretakers who may also be living with HIV. DPP is located in the Dar es Salaam city center and serves a mixed urban and rural population, with a total patient enrollment of approximately 285 children and 162 parents/caretakers.

### Enrollment procedures

The DPP clinical staff identified a convenience sample of children living with HIV who were eligible for IPT according to national guidelines during the study enrollment period. Specifically, eligible patients were 1) HIV-infected, 2) between the ages of 1 and 15 years, 3) enrolled in care at DPP, 4) ruled out for TB disease by using the National Tuberculosis and Leprosy Program (NTLP) symptom screen tool, 5) without contraindications to IPT as per national guidelines (e.g. severe liver disease or known adverse reaction to isoniazid), and 6) not planning to move outside of Tanzania in the next 6-months. Per NTLP guidance, only children who had signs or symptoms for TB based on the symptom screening tool were required to undergo a chest x-ray and sputum testing. If a child had a negative chest x-ray and sputum testing, they were eligible to participate in the study. Sputum testing involved AFB smear, culture, and Xpert MTB/RIF. The study nurses obtained verbal informed consent in Kiswahili using a standardized form, which included counseling on the risks and benefits of IPT. Informed consent was obtained from parent/caregivers. Children who were able to (typically those over age 5) provided verbal assent to a nurse who used age-adjusted language and questions to gauge the child’s understanding. The parent/caregiver, in conjunction with the child (when they were old enough to participate in the decision-making), then selected their IPT delivery model and were dispensed isoniazid (INH) (dosed at 10-15 mg/kg up to a maximum of 300 mg/day). Most children received a 1- or 2-month supply of INH, based on the timing of their next scheduled appointment for ART.

### IPT delivery models

Children (and their parent/caregiver) were offered a choice of either facility- or community-based IPT delivery models. In the facility-based model, TB screening, adherence assessment, and INH refills were coordinated with the child’s routine ART refill appointments at the clinic, typically in monthly or bimonthly intervals. Roundtrip travel to the clinic via public transportation was reimbursed for all children and their parents/caregivers regardless of study involvement. One clinician and two nurses performed the screening, adherence assessment, refills, and follow-up of patients. In the community-based model, nurses performed the same functions in the child’s home and dispensed both their INH and ART refills. Patients could switch models at any time during the study and were reminded of this at each follow-up appointment.

### Follow-up visits and monitoring

IPT refill intervals followed the child’s pre-existing ART refill schedule. The study nurses attempted to contact by telephone any study participant who missed an appointment as per routine clinic practice. Adherence was assessed at each visit and IPT completion was documented after the final IPT refill. Average monthly adherence was determined via pill counts. Follow-up visits were attended by the child and parent/caregiver, the child alone, or the parent/caregiver alone. Regardless of who attended the follow-up appointment, INH pill bottles were examined for adherence assessment. INH and ART refills were provided at each follow-up visit. In addition, participants were screened at every appointment for TB disease using the NTLP-recommended symptom screen. If any TB signs or symptoms were present, a chest x-ray and sputum for AFB smear, culture, and Xpert MTB/RIF would be performed. Participants with confirmed TB disease were referred to a separate TB clinic for treatment; if TB was excluded, the child resumed IPT.

### Outcomes

The primary outcome was IPT completion, defined as completing a 6-month course of INH within 10 months. When exact treatment completion dates were not documented, the completion date was estimated by adding the days of IPT remaining (as indicated by the number of pills that the patient had remaining) at the time of their last IPT appointment. The secondary outcome was adherence, defined as the percentage of prescribed INH taken. Adherence was measured using a combination of pill counts at follow-up appointments, appointment adherence, and patient self-reporting. Pill counts were obtained at each follow up visit and used to calculate monthly adherence. Appointment adherence was used to supplement pill counts by calculating how many INH pills should be left given the interval between appointments. Rarely, participants provided additional information to explain why the pill count and appointment attendance might not accurately reflect their adherence, such as when they lost or dropped pills, to allow our study team to make corrective calculations to their adherence.

### Training and monitoring

One clinic physician and two nurses were trained in IPT administration. The study team conducted staff training through didactic lectures, discussion, and role-play. Training sessions focused on 1) review of the national IPT policy, 2) review of the benefits of IPT, 3) results from the IPT program evaluation, 4) patient motivational interviewing techniques and shared decision-making, 5) overview of the project using the approved standard operating procedures, and 6) transfer of data collection tools.

### Data analysis

Study data were collected using standard paper registers. Basic demographics, method of IPT delivery, reasons for choosing that delivery method, pill counts, dates of IPT initiation and refills, adverse reactions, deviations from prescribed treatment, and patient-initiated switches from the initially-selected delivery model were all recorded. Qualitative data notes, such as reason for choosing a delivery model, were documented by the study staff on the register and later categorized as: general preference (reason not specified), confidentiality/stigma concerns, ease, perceived better treatment support, and other. These data were transferred to a password-protected Microsoft Excel 2010 spreadsheet on password-protected study laptops. Data were exported to JMP v13 for final analysis. All data were validated by comparison between paper and electronic entries. Descriptive analyses using means/medians and proportions were conducted for continuous and categorical data respectively. Adherence was calculated at the first follow-up and for all subsequent visits with available data.

The study protocol was approved by the National Institute for Medical Research in Dar es Salaam, Tanzania; Muhimbili University of Health and Allied Sciences (MUHAS) Institutional Review Board in Dar es Salaam, Tanzania; and the Dartmouth Committee for the Protection of Human Subjects in Hanover, NH, USA.

## Results

We enrolled 66 eligible children between July and December of 2017, and the last child finished IPT in July 2018. Although not formally documented, the clinic nurses reported that out of the 66 children approached, all accepted IPT. Most participants were female (*n* = 43, 65.1%) and all were on ART. The median age was 11 years (interquartile range [IQR] 8–13). Most children did not have a history of TB (*n* = 54, 81.8%) and 33 (50.0%) were at WHO clinical stage III for HIV at initiation. Detailed participant characteristics are shown in Table 1.

**Table 1:** Characteristics of 66 Pediatric Participants Receiving Isoniazid Preventive Therapy

Characteristic	Total (*n* = 66) *n* (%)	Summary Statistic
Sex		
Female	43 (65.1)	
Age at Initiation, median (Q1, Q3)^1^		11 (8, 13)
0–5 years	6 (9.1)	
6–10 years	21 (31.8)	
11–15 years	39 (59.1)	
WHO Clinical Staging for HIV at Initiation		
I	3 (4.5)	
II	26 (39.4)	
III	33 (50.0)	
IV	3 (4.5)	
Missing or not reported	1 (1.5)	
History of Tuberculosis		
Yes	12 (18.2)	
No	54 (81.8)	
ART Status		
On ART	66 (100.0)	
Duration of ART at initiation in years, median (Q1, Q3)		5 (2.25, 8)
History of Liver Disease		
Yes	0 (0.0)	
No	57 (86.4)	
Missing or not reported	9 (13.6)	

^1^(First Quartile, Third Quartile)

The facility-based model was selected by 63 (95.5%) participants at IPT initiation. (Table 2). Most cited confidentiality concerns (*n* = 30, 45.5%) as their main reason for choosing the facility-based model, specifically because they feared that the community-based model would inadvertently disclose their HIV status to their neighbors. Other reasons for choosing the facility-based model included ease (*n* = 9, 13.6%) as visits to the facility for HIV-related care were already integrated into their routine, and a general preference to maintain their routine (*n* = 18, 27.2%). Most participants (*n* = 61, 92.4%) retained their initially chosen model throughout the study. Of the 5 participants who switched models, 3 (60%) switched once after the first IPT visit, and 2 (40%) switched multiple times throughout the study. Of the 5 participants who switched their models (either once or many times), 3 (60.0%) initiated the study by selecting the facility-based model and 2 (40.0%) initiated with the community-outreach model.

**Table 2:** Patient Selection of Facility-based versus Community-based IPT Delivery Model

Characteristic	Total (*n* = 66) *n* (%)
Model at Initiation	
Facility-based	63 (95.5)
Community-based	3 (4.5)
Model Consistency	
Maintained chosen model	61 (92.4)
Switched model once	3 (4.5)
Switched models multiple times	2 (3.0)
Reason for Model Choice	
General preference (unspecified)	18 (27.2)
Protect confidentiality	30 (45.5)
Easier for patient	9 (13.6)
Better treatment support	2 (3.0)
Other	4 (6.1)
Missing or not reported	3 (4.5)

### Adherence and treatment outcomes

Forty-nine (74.2%) participants were confirmed to have completed IPT. Of those that completed IPT, pill count data showed a 98% average monthly adherence. Only one child had an adherence of less than 70%, and 33 (50%) had adherence rates between 93 and 100%. The recorded monthly adherence of 98% is well above the 80% standard threshold for adherence. The estimated mean time to complete IPT was 27.6 weeks (6.4 months) among the 35 participants for whom a precise completion date could be determined. Incomplete record keeping meant that not all participants had an exact date of IPT completion. All participants who completed their IPT did so within 10 months of initiation, and no participants missed more than 90 days of IPT doses. Data on IPT adherence and treatment completion are shown in Table 3.

**Table 3:** Adherence rates for 49 patients who completed treatment with adherence data confirmed by pill count

Characteristic	Total (*n* = 49) n (%)	Summary Statistic
Average monthly adherence, median (Q1, Q3) ^1^		98.0% (93.0, 100%)
< 50%	0 (0.0)	
50–79%	1 (2.0)	
80–100%	45 (91.8)	
Missing or incalculable	3 (6.1)	
Days of IPT missed, median (Q1, Q3)		11 (1, 33)
Approximate duration of IPT (weeks), mean (range) ^2^		27.6 (20–38)

^1^(First Quartile, Third Quartile)

^2^This value could only be estimated in 35 participants

Among the 17 (25.8%) children who were not included in the adherence analysis, 5 (29.4%) were lacking IPT completion documentation in their medical records, 11 (64.7%) discontinued treatment, and 1 (5.9%) had an unknown outcome due to missing paperwork. Inconsistent documentation practices and patients forgetting to bring their pill bottles for adherence review (pill counts) resulted in incomplete IPT documentation that prevented adherence determinations in some cases. Among the 11 participants who discontinued IPT, 7 (63.6%) did so at the recommendation of the physician due to one or more adverse reactions including joint pain (*n* = 2), vomiting (*n* = 2), numbness (*n* = 3), oral ulcers (*n* = 1) and rashes (*n* = 1). No cases of TB were suspected or confirmed among participants while on IPT or during a median of four additional months of follow up.

## Discussion

Our study findings demonstrate that high rates of IPT adherence and treatment completion are achievable in a pediatric HIV clinic population through IPT delivery coordinated with ART refills. While too few patients selected the community-based model of delivery to compare the effectiveness of the two models, the overall model of self-selected IPT delivery led to high adherence across the entire study cohort. Integrated care, achieved by pairing IPT with ART refills, meant that no additional medical visits were necessary and subsequently no additional burden was incurred for the child and their family. Maintaining routine care patterns likely contributed to the high adherence outcomes, as many patients cited this as a reason for choosing their delivery model. Of the six children who were not included in adherence analysis, 5 (83.3%) are believed by the clinic staff who administer their HIV care to have completed IPT but simply lacked official documentation. Among these 5, the mean estimated IPT completion date was 6.5 months after initiation. Thus, it is likely that these five children completed their IPT within the 10-month limit, even if their files did not specify an end date. Using these estimated completion dates, a total of 81.8% (54/66) completed their IPT within the specified timeframe.

Where data were available, adherence was very high (98% monthly average). The fact that this study examined an “ART-established” cohort of participants (with all but one participant having initiated ART > 1 year before IPT initiation) likely contributed to the excellent adherence as participants were habituated to taking daily medication. Our study parallels other similar findings in IPT adherence. Previous studies have found high adherence to IPT amongst pediatric populations living with HIV, suggesting that IPT is a feasible intervention for wider implementation [21, 26]. This study contributes to the adherence literature by examining IPT delivery models within Tanzania. Shared decision making and a patient-centered approach to care has been shown to improve child/patient satisfaction with their healthcare and increase medication adherence [27]. Furthermore, the WHO’s “Framework on integrated, people-centred health services” recommends the implementation of five strategies to improve equitable access to care: (1) empowering and engaging people and communities, (2) strengthening governance and accountability, (3) reorienting the model of care, (4) coordinating services within and across sectors, and (5) creating an enabling environment [28]. Our findings align with this framework by engaging individuals and families as active participants in their care, creating an equal and reciprocal relationship between clinical professionals and individuals, and individualizing self-management and treatment plans [28].

Concerns regarding breakthrough TB disease in the pediatric population were not borne out in this study. There were no cases of breakthrough TB and no presentations that warranted further evaluation with bacteriological investigation. The lack of breakthrough TB supports the use of the NTLP-recommended symptom screen to determine patient eligibility for IPT. INH was well tolerated by the majority of the study population. 16.7% (*n* = 11) of children stopped IPT, primarily due to minor adverse reaction. Shorter regimens to treat latent TB infection have been shown to be as effective as IPT, with high adherence, and potentially better tolerability, including 3 months of daily INH and rifampin, 3 months once-weekly INH and rifapentine, and 4 months of daily rifampin [29, 30]. Future studies should apply the patient-centered delivery choice in this study to these additional regimens.

Our study had three main limitations. First, data on adherence were limited due to incomplete data capture of pill counts by nurses and inconsistent presentation of pill bottles by participants at every appointment (to enable accurate adherence calculations). Similar difficulties in record keeping for IPT delivery have been documented in other similar studies in the region [31]. Nonetheless, while pill count data were incomplete for individual patients, the aggregate data indicate that IPT adherence was consistently high in this pediatric population. Furthermore, a recently published study by Martelli et al. (2019) [32] on pediatric ART adherence in Tanzania found that there was a large lack of agreement between pill count/observed adherence data and adherence data derived from the patient’s viral load (39.9%), suggesting that pill counts may not be an accurate indicator of adherence. Other methods of adherence should be considered for future studies, such as urine analysis to assess INH metabolites, to provide additional objective adherence data [33]. Second, because medication could be dispensed to the child or the parent/caregiver, sometimes the child was not present at follow-up visits. While the study team did not capture the rate at which parents/caregivers attended the appointment alone, this was uncommon. A flexible follow up and INH refill model may have increased appointment adherence as it allowed for pills to be counted and refills to be dispensed even if either the child or the parent/caregiver could not attend; however, this flexibility meant information on TB symptoms and adverse reactions could be provided secondhand by the parent/caregiver, potentially compromising accuracy. Third, the size of the cohort was relatively small. Such a small sample size may have limitations in the scalability of the study’s findings. Further studies with larger cohorts would increase the generalizability of our findings.

Our study demonstrates that high adherence and treatment completion rates can be achieved when IPT is integrated into pediatric HIV care and when patients and their parent/caregiver are involved in choosing delivery methods that best fit their needs. Improved record-keeping and patient pill bottle presentation at appointments may yield even higher completion rates. There were no detected cases of TB. For children living with HIV, IPT can be implemented effectively and should be recommended throughout Tanzania.

## Conclusions

Our study demonstrates IPT feasibility and high completion rates among children living with HIV in Tanzania. When flexible delivery models and shared decision making are included in standard care, high adherence and treatment completion rates of IPT can be achieved. Coordinating IPT and ART is an effective way to minimize patient visit burden and streamline care delivery. There were no cases of TB were detected during or after the study. Improved record-keeping may yield even higher completion rates. IPT should be implemented into routine pediatric HIV within Tanzania.

## Data Availability

The datasets used and/or analyzed during the current study are available from the corresponding author on reasonable request.

## Abbreviations

ART: Antiretroviral Therapy
INH: Isoniazid
IPT: Isoniazid Preventive Therapy
HIV: Human Immunodeficiency Virus
TB: Tuberculosis
WHO: World Health Organization
