# Correction to: Successful implementation of isoniazid preventive therapy at a pediatric HIV clinic in Tanzania

**DOI:** 10.1186/s12879-020-05514-5

**Published:** 2020-10-20

**Authors:** Olivia F. Hunter, Furaha Kyesi, Amrit Kaur Ahluwalia, Zeinabou Niamé Daffé, Patricia Munseri, C. Fordham von Reyn, Lisa V. Adams

**Affiliations:** 1 Dartmouth College, Hanover, NH 03755 USA; 2 New York, USA; 3 Ministry of Health, Community Development, Gender, Elderly, and Children, Dodoma, Tanzania; 4 Muhimbili University of Health and Allied Sciences, Dar es Salaam, Tanzania; 5 Dartmouth-Hitchcock Medical Center, Lebanon, NH 03756 USA; 6 Geisel School of Medicine at Dartmouth, Hanover, NH 03755 USA

**Correction to: BMC Infect Dis 20, 738 (2020)**

**https://doi.org/10.1186/s12879-020-05471-z**

Following publication of the original article [1], the author identified an error in the author name of C. Fordham von Reyn.

The incorrect author name is: C. F. von Reyn.

The correct author name is: C. Fordham von Reyn.

The author group has been updated above and the original article [1] has been corrected.
